# An Analysis of Guislain's Work on Insanity

**Published:** 1855-07-01

**Authors:** 


### AN ANALYSIS OF GUISLAIN'S WORK ON INSANITY.

### Twelfth Lecture.

(*Continued from No. XXVII., page* 443.)

*On delirium or disorder of the ideas.*—I have shown you the moral sensibility painfully affected, the passions diseased, and the pathological perturbations of the will. Now let us consider morbid ideas, delirium. Delirium, which I shall define to be a marked aberration of reason, is an error in the conceptions, a disorder in the ideas, which the patient can neither resist, nor put an end to; it is always a chronic condition in which he regards as realities the phantoms of his imagination.

The delirium may be *general*, or partial when it relates to certain isolated ideas. There are two species of delirium—one *essential*, pure, constituting a disorder absolutely simple; the other *symptomatic*, secondary, tertiary, arising at the same time as other disorders, and disappearing with them. In special delirium, the patients preserve more or less the aspect and bearing of a healthy man. The memory remains intact; they count, calculate; they distinguish right and wrong; they judge of events; up to a certain point they can conduct themselves suitably in society, sometimes even carry on their affairs. Most frequently delirious madmen are not conscious of their state; they look upon their dreams as realities. There are states in which reason and the imagination engender errors, and in which the patient feels that he is the sport of an intellectual phantasmagoria. This state is not delirium.

In partial delirium, the sleep may be disturbed; but the nutritive functions are rarely disturbed.

I recognise four distinct categories of erroneous conceptions,—1. An *accusative delirium*.

Many patients thus affected talk of secret means which their pretended enemies use against them. Often these imaginary beings act at a distance; they have electricity, magnetism at their disposal.

A captain, formerly aide-de-camp to Byron, in Greece, now in this establishment, is convinced that enemies in the island of Ipsara work upon his mind by the aid of a machine he does not describe. "Yes, sir, those villains yonder are working the machine . . . . you know the machine." If you ask him what machine? he smiles cunningly, as if to say, "You, too, are setting a trap for me."

We have patients profoundly impressed with the belief that the pump-water is poisoned, and that arsenic is put into all the food. They refuse food accordingly. In this case the refusal has a motive; they refuse because they think you want to get rid of them. In folly, on the contrary, this refusal is a caprice of the will; the patient refuses to eat without knowing why.

Others behold spies on every side. The features of these change, they turn pale at the sight of another patient, or of a keeper approaching them; they take them for assassins or traitors. This species constitutes a form of transition which connects delirium with mania; the whole condition announces excitation, exaltation. In simple accusative delirium, the patient is much more calm.

2. The delirium of *inspiration*. I define the condition of those affected with this form by describing them under the term of *erotic monodelirious*, religious, ambitious, and *hypochondriac monodelirious*.

Erotic acts are sometimes accompanied by a marked derangement in the conceptions and ideas. These are false interpretations, pretended marriages, the persuasion of having had children contrary to the fact. This is metromono delirium.

Religious monodelirium takes the forms of theomonodelirium, monodemonodelirium, monodemonolatry, when the patient persuades himself he is in hell, and worships Satan. This vesania, in our days very rare, was common, and sometimes epidemic in the fifteenth and sixteenth centuries.

Religious delirium also includes prophets, men who believe they enjoy a celestial existence, and madmen calling themselves God. You will observe in examining these patients, that you must excite them in the direction of their delirium, in order to bring out their delirious conceptions. They may reason well upon a number of subjects, provided you do not touch upon that of their delirium.

We have also kings, queens, and princes.

There is a class of delirious illusionists whom I will call the metamorphosed. There is a patient who believes himself transported into a dwelling which is not that where he really is. There is another who believes he has grown a foot in the course of the night; all his teeth have fallen out, but he pretends to have got others, much whiter; he has no longer legs, or arms; his legs are glass; he has animals in his stomach; he has no bowels.

Under this order of vesania must be classed the zoanthropists, who imagine themselves changed into beasts.

A fourth group comprises those labouring under hallucinations. I have met with women who cried out for help, imagining that their children were being butchered in the adjoining room. In others the hallucinations are visual. Others fancy they smell foul odours. Less frequently the sense of taste or that of touch is affected.

But these forms are not often simple; several forms are observed together.

Intermissions are less perceptible in delirium than in mania. The duration of this kind of alienation is very long. It may last for years without much affecting the health. Sometimes it ends in dementia; rarely in mania.

### Thirteenth Lecture.

### Of Dementia, *or the obtusion and obliteration of the phrenic acts.*

*The phenomenology of dementia.*—Dementia is the weakening or obliteration, more or less complete, of the moral and intellectual faculties, often accompanied by the diminution or extinction of the motive power.

Five fundamental types compose this kind of vesania:

*Pure dementia:* the exhaustion, more or less general, of the phrenic faculties.

*Stupidity:* the partial or total suspension of the intellectual and motor acts.

*General paralysis:* the progressive paralysis of the moral, intellectual, vocal, and locomotive phenomena.

*Imbecility:* the imperfect development of the moral and intellectual faculties.

*Idiocy:* the non-evolution, or defective evolution of the mental faculties, accompanied most frequently by disorder in the locomotive acts; a disease connected with a congenital state.

This division is based upon the phenomenology. Pinel, Esquirol, and many other physicians have adopted a division drawn from the symptoms. They distinguish dementia from idiocy. But it is impossible to see in idiocy, if we look to the morbid form, anything but a variety of dementia.

I admit a dementia pure, false, complete, incomplete, special, general, primary, consecutive, simple, compound.

1. *Pure dementia: amentia, fatuitas, anoia.*

Dementia contrasts strongly with the vesaniæ we have hitherto considered. It is an exhaustion, a falling-back of the phrenic powers. The patient hears, sees, and cannot distinguish; he neither understands nor appreciates. Dementia is announced by an expression of subjection, of apathy, of intellectual nullity, by an attitude relaxed and indolent, a want of dignity, a certain incapacity for bodily movements, a slow elocution, inane, childish, or unmeaning answers, a difficulty, an impossibility of forming ideas, a stupid and indifferent bearing.

Dementia is *false* or *real*. There is a condition which often deceives the world. Whenever an insane person has no spontaneity, ceases to recognise and to understand, remembers no longer, he is looked upon as an imbecile. Well! these are not the true elements. In such a man there may be an oppression, and not an extinction of force. This is especially applicable to acute melancholy and acute mania, in which diseases the intelligence seems to be covered with a veil.

This is the acute dementia of some authors.

Dementia may be *complete* or *incomplete*.

In the first case, the mental faculties are dead. Dementia is incomplete when the patient recognises the members of his family, remembers the name of the street he lives in, when his evacuations are not involuntary. The English call this "apathetic insanity." Recovery is not hopeless. I have met with it in men recently married, in drinkers, in epileptics, taking the place of convulsive attacks, and after long sorrows.

Another variety of intellectual decadence has been called *hebetudo psychica;* it consists especially in the weakening of the judgment, of the reason.

There are *special* dementias, and *general*. The patient may experience a considerable impairment in a certain range of his phrenic faculties; he may be in a state of *monodementia*, preserving the remaining faculties intact. Thus he may retain an artistic talent, remain a good painter, a good musician. There is a phrenopathic condition which consists simply in loss of memory.

In another class of vesaniæ opposed to those I am speaking of, the patient performs tolerably well certain acts, but he is affected with extreme disorder in his speech. This is *incoherence of ideas*.

Dementia is *primary or secondary*. When primary, it is closely linked to the causes from whence it sprang. This is especially the case when the disease is associated with debilitating influences, as the abuse of drinks, spermatorrhœa, advanced age, antecedent sickness, penury. It is also primary when moral causes re-act upon delicate, feeble subjects. During the last three years we have entered an exorbitant number of cases of primary dementia.

Dementia is secondary when it follows upon melancholy, mania, folly, delirium, ecstasy.

Sometimes dementia is a *compound* disease. An elementary dementia, strictly speaking, is rarely met with. Thus there is dementia with mania, the desire of incendiarism, disposition to suicide, to homicide, with automatic gestures.

Generally, the gastric organs perform their functions with regularity in dementia. Sometimes, however, deglutition is impeded. The pulse is feeble, and preserves an acceleration we have observed in the other kinds of alienation. Frequently we remark an abundant accumulation of fat. Dementia follows a progressive course, during which the degradation of the intellectual faculties is seen to be gradually affected, until at length the patient falls into a state of more or less complete moral annihilation.

Sometimes, however, mania succeeds to dementia, which undergoes a transformation.

Commonly, dementia ends in cerebral marasmus, in a peculiar condition only seen in the insane. The patient wastes away, his body becomes bowed, atrophied, and anchylosed; lying in bed, his knees are in the air, his head scarcely touches anything. Intelligence goes first, then instinct, and the man ends in being no more than a stomach, calling in vain upon the cerebral centre for assistance. Dementia may go on for many years, but from the moment that cerebral marasmus appears, a few months, or weeks, carry the patient to the grave. Death mostly comes on suddenly.

*Stupidity.*—As to its form, this condition resembles the other kinds of dementia. It differs essentially, in offering a great prospect of recovery. It has been regarded as an elevated degree of melancholy, a phrenalgia passed over into a state of dementia. This would be the *melancholia attonita* of ancient pathologists. This view is perhaps not far wrong.

### Fourteenth Lecture.

*General paralysis.*—The patient now brought to us presents a look that expresses astonishment. See his smile of imbecility, his faltering gait! He is affected with general paralysis. His age is thirty-four. His wife is twenty-one. His life has been marked by great excesses. He is a cooper, employed in a brewery. He was habitually given up to drunkenness and debauchery. He has not been happy in his home. His attitude betrays a loss of equilibrium; in walking, he spreads out his legs, and carries his arms out and his head backwards. I will speak to him. You will remark in his answers, a hesitation quite characteristic in the formation of words and phrases. . . . . He does not understand what we say to him. He sees, but he does not look; he does not conceive in seeing. He no longer recognises anybody. His discourse is marked by strong exaggeration. He is subject to gusts of passion; he excites himself, and complains. He has delirious ideas; he thinks everything belongs to him; he talks of his fine clothes, his beautiful wife, his handsome chairs, his goblets of crystal.

The disease has been preceded by a long period of incubation, marked by weakening of the phrenic faculties. Then symptoms of delirious mania appeared. From the commencement, a slight hesitation was observed in his speech, a certain tension was noticed in his neck, a fixedness in his look, a *facies* quite peculiar betrayed to the eye of the practitioner the gravity of the case.

Generally, the course of this disease is marked by two orders of phenomena, permanent and transitory. The first consists in gradual failing of conception, memory, and all the phrenic faculties; the others in outbreaks, effervescence, crises, fits appearing at various intervals, and which, after having first manifested themselves by rigidity, entail relaxation of the muscles, paralysis, finally convulsions and sopor.

In this disease one pupil is at times more dilated than the other. M. Baillarger has described this as a new symptom of general paralysis. But it also belongs to mania.

In general paralysis it is not so much the *strength* of the movements that is lost, as their *precision*, as Foville has well remarked.

As the paralysis of intelligence and motion advances, sores form on the back; frequently the patient gives no sign of suffering, but fever consumes him. A comatose state supervenes, epileptiform convulsions appear. The pharynx becomes paralysed.

General paralysis is rarely an acute condition. It is a chronic disease which may terminate in the course of a year, but which may last two, three, five years. Most frequently the patient finishes in the second year.

Several questions present themselves touching the pathology.

Is paralysis of motion the radical symptom of the disease?

Does the phrenic psychical state succeed the disorder of motion?

Is the phrenic state, the moral intellectual disorder, primitive; and is the paralysis of motion the consequence of the first state?

Is then general paralysis, without perturbation, a marked failing of the psychical state?

Is there psychical paralysis without muscular paralysis?

I answer, that no one of the phenomenal groups of general paralysis has a constant priority in the order of development of the disease. These phenomena by turns predominate, muscular failure, intellectual failure, delirium of ideas. Each of these elements may have a maximum or a minimum of value in the course of the disease.

The most initial of all the paralysiform symptoms is the hesitation of speech; but it is not always the first.

Latterly M. Baillarger has communicated facts which prove the importance of the basis of the movements.

M Lunier adduces facts to show that general paralysis is a distinct disease from mental alienation. M. Moreau considers the physical symptoms and the psychical symptoms as belonging to the same source. I can call to mind cases which exhibited the distinction between the physical and psychical symptoms.

Is the disease ever secondary? is it always primary?—In the great majority of cases it is primary. I do not remember even to have seen it occur as an accidental symptom in the course of melancholy, as a consequence of ecstasy, or of a destructive phrenopathy. But I have observed it, occasionally, as an epiphenomenal termination of delirious congestive mania.

The general paralysis of the insane must be distinguished from *apoplectiform paralysis.*

IMBECILITY, *amentia, morosis* of Sauvages.—The imbecile have not lost their intelligence; the faculty is only weakened, imperfect. The imbecile have become such after birth; they could never learn to read or write, or a trade; they express themselves with tolerable correctness; but judgment is wanting, and very few of them have memory.

Imbecility is frequently associated with other states, especially with vices of character, or attacks of mania. Many imbeciles are thieves, many quarrelsome, mischievous, &c. It is rarely associated with delirium.

IDIOCY is a congenital dementia in which the degradation of the intellectual faculties is such as to debase man below the brute, even below the plant, since all the functions are so lowered, that without the assistance of another person, some idiots would be incapable of feeding themselves.

Most modern authors have made a distinct genus of idiocy. I do not see the necessity of this. I therefore include it under the genus *amentia, dementia, secordia, fatuitas, paranoi* of the Greeks.

Idiocy is frequently associated with epilepsy; sometimes with paralysis, or muscular atrophy. M. Ferrus has established a distinction between idiocy and cretinism.

Imbecility and idiocy have an especial interest in relation to legal medicine. Imbeciles and idiots frequently figure before courts of justice, accused of outrages against decency, of theft, arson, and murder.

### FIFTEENTH LECTURE.

*Of the manner of considering the organic alterations which present themselves in mental diseases. The anatomical diagnosis.*

*How identical cerebral symptoms may indicate diseases of different nature.*—The anatomical diagnosis comprises, 1st, the knowledge of the phenomena supplied by opening the body; 2nd, the signs which indicate in the living body the changes in the brain and other organs. Mental alienation does not mean what is called a disease of the brain, a disease of the encephalon. Cerebral diseases may manifest themselves without mental alienation; and this may exist without cerebral disease. In either case, there are often presented identical phenomena. Art should consist in determining whether this symptom be a functional disorder, whether that, announce an anatomical lesion. Most frequently alienation is a functional affection; but this latter may induce a cerebral disease. The symptoms which indicate a *cerebral disease* are, incoherence, the delirium of ideas, impairment of conception, loss of memory; coma vigilans, coma, sopor especially; loquacity, gesticulations; more or less general tension of the muscular system; a great prostration; singing in the ears; vertigo; pains in the head, in the limbs, a painful condition of the skin, formication; nausea, vomiting, dilatation, contraction of the pupils.

Now, incoherence, sopor, disorder of the intelligence, have a quite different signification in mental diseases to that which they possess in affections of the brain, in febrile diseases, and in nervous affections and intoxications. In the insane, delirium is far from indicating an inflammation of the meninges; stupor is by no means connected with inflammatory or purulent congestion. In cerebral diseases, there exist direct relations between the cause and the effect more appreciable than in mental diseases. In the latter, the action of the anatomical element escapes us.

The idiopathic phrenopathies are the only diseases to which it is proper to give the name of mental affections; they have especial origin, course, and phenomena. We must admit these fundamental species of mental diseases; idiopathic phrenopathies; symptomatic; and sympathetic phrenopathies.

It follows, that in order to be a mental physician, your practical knowledge ought not to be limited to the insane; you ought not to be a speciality in the rigorous acceptation of the word. I cannot tell you often enough that the way to make progress in the study of the phrenopathies is to call to your aid the general notions of the theory and practice of medicine. It is especially when the question of the diagnosis and treatment of mental affections arises, that the necessity of having seen many insane and many patients of other kinds is felt in all its force.

*Cerebral alterations which present themselves in mental diseases; the symptoms by which they may be recognised.*—I reduce to the number of nine the lesions of the encephalon, to which I wish to direct your attention. These are: 1, sanguineous congestion of the meninges, of the brain, of the meninges and brain. 2. Serous congestion of the same structures. 3. Cerebral softening. 4. Opacity, thickening of the Arachnoid. 5. Meningeal and cerebro-meningeal adhesions. 6. Cerebral induration. 7. Cerebral hypertrophy. 8. Cerebral atrophy. 9. Faults of conformation of the brain and skull.

Even this number may be greatly reduced. The essential conditions are: sanguineous and serous congestion, softening, induration.

Congestion occurs under two different forms. It may be active, arterial; it may be an inflammatory state, or closely approaching; or it may be passive, venous. The active state declares itself in alienations, characterised by violent reactions. But you must not believe that the brain is congested every time that the phrenic disorder is announced by the violence of the passions. Four times out of five, the most turbulent mania is not accompanied by a true congestive state. The most fearful errors are committed in this respect.

*Diagnosis of cerebro-meningeal determination.*—The symptoms which give rise to uneasiness in the physician are: the persistence of the disease; the increase of the disorder in the ideas; the complete absence of days of calm and lucidity; the resemblance of an acute delirium in a chronic case; confusion, incoherence of ideas, proceeding side by side with the decadence of conception and memory. There is a veil stretched over all the conceptions.

What marks more purely congestion is: 1st, the robust plethoric constitution of the subject; the injection of the face; a certain brilliancy of eye; a strong heat on the surface of the cranium; the febrile frequency of the pulse; sweats, often clammy, bathing the surface of the head; ammoniacal, hypostatic urine; an air of astonishment, a deafness, a blindness of the intelligence, drunken ideas.

What marks especially congestion, inflammatory fluxion of the brain and meninges, is: the agitation of the patient; the stiffness of his limbs ; disorder in his muscular acts; prostration; involuntary evacuations; a dementia which follows mania; convulsions: paralysis. Rarely, however, the symptoms express a fierce inflammatory state, and end quickly. Generally the disease assumes the chronic form.

Sudden abolition of the faculty of speech, of all the faculties of the intelligence, denotes compression of the cerebral surfaces. These patients present a false appearance of apoplexy; but true paralysis is wanting: the eyes remain open, and the patient can move his limbs. Automatically the patient carries his hand to his head, which seems to suffer shocks; it is tossed from side to side; sometimes there is grinding of the teeth, distortion of the features, stiffness of the limbs. Sometimes vomiting announces a rapid and fatal progress.

These symptoms may give way under appropriate treatment. In speaking of crisis, of treatment, I shall take care to show you that recovery is sometimes preceded by a febrile, comatose state: this must not be confounded with that condition which may be the effect of a congestive inflammatory orgasm of the meninges or of the brain. In this state there are stages: first, a period in which the ideas give a colour to the passions: so long as these are clear, though extravagant, there is no fear of congestion and its consequences. To this period succeeds a phase of obscuration of the ideas and of disorder in their manifestation. A third period marks the gradual extinction of the faculties of the understanding.

Bayle first called attention to the relation between notions of greatness and the congestive state of the meninges, and the cortical substance of the hemispheres.

In the presence of this group of symptoms, we may believe that a congestion is forming on the surface of the brain; and this especially when the subject has been addicted to alcholic drinks. You will also meet with it in persons become insane from the action of the solar rays on the skull, or under the influence of intense radiant heat. It is observed in cases of retrocession of an exanthema.

When congestion leads to sanguineous effusions between the meninges, the symptoms are usually very alarming. They are characterised by a sudden change in the physical and moral state of the patient. First, a comatose state, then a sensible diminution in the sum of the intellectual acts. At other times, a complete hemiplegia, or convulsions.

In drawing the diagnosis of these congestions, of these orgasms, of these spinal inflammations, take care not to perceive in the phenomena which characterise them the whole disease. I shall tell you by and bye that mental alienation is not in its intimate nature a congestive state, an inflammation. Inflammation may be developed in alienation, it may be strictly associated with the first state; but it is not the sum of the mental affection.

If an epileptic maniac dies during a fit, we may be almost certain to find a state of red congestion of the meninges and of the cerebral substance, even ecchymosis, blood extravasated in the tissue of the membranes, especially in the temporal regions. If the epileptic die during an interval, nothing of the sort is found. So it is with alienation; the congestive state is subordinate to the exaltation of the intellectual phenomena.

Out of five hundred patients congregated here, I cannot at this moment show you one case of fluxionary congestion in its first phase.

*Venous or black congestions.*—I believe that there are amongst the insane venous congestions, independently of the congestions which proceed from a nervous orgasm. Cases of venous hyperæmia are frequent in the dementia following upon chronic mania. It is especially when the patient has vociferated much that the cerebral substance is found gorged with black blood. In melancholy, we find sometimes the sinuses and veins of the arachnoid strongly congested; but it is rare to find an active congestion in these patients. Do not lose sight, also, that the congestion is at times only apparent, and that it depends upon cerebral hypostasis formed in the last moments.

The congestional state is a very frequent symptom in general paralysis; out of 25 cases, it is met with at least 11 times.

*Microscopical examination.*—I have committed to the microscope cerebral substance congested and not softened, and I have satisfied myself that the anatomical result of congestion consists in a cellular development. It seems as if the primitive cells constituting the intimate web of the brain undergo a certain distension; that they swell from the presence of a fluid. There is a remarkable difference between congested cerebral substance and that which is not: in the first, the field of the microscope is covered by a stratum of granulated matter interspersed with corpuscles, which I conclude to be fatty, since they dissolve in ether. I will state, with reference to these corpuscles, that they are seen in the healthy brain as well as in the diseased.

*Serous collections.*—We meet in the insane with serous accumulations in the cavities of the membranes and in the ventricles. It is principally the pia mater that is œdematosed; the œdema is united with a venous congestion. The serosity is more frequently gathered between the meninges than in the ventricles. Sub-arachnoïdean collections are especially common.

Recently there has been discovered an œdema seated in the brain itself. MM. Foville and Ferrus were the first to speak in precise terms of an interstitial infiltration of the brain. Esquirol, it is true, had mentioned it. M. Estoc has studied this condition with great care, pointing out the kind of alienation in which it is most frequently seen.

The origin of serous collections is in many respects an enigma in the study of mental diseases. It must be concluded that most commonly they depend upon venous congestion. But frequently we find, instead of a red injection of the vessels, a true anæmic condition of the cerebral substance. In many cases of chronic dementia, serous collections are formed when the shrunk brain falls away from the internal table of the skull. Magendie's experiments seem to explain the formation of an intra-cranial fluid whensoever a vacuum is formed between the surface of the brain and the internal surface of the cranium.

*Diagnosis.*—Here is a patient who, I have been told, is affected with stupidity. I exhibit him to you again, in order to point out the symptoms, or rather the appearances which announce the presence in the brain of an excess of serosity, infiltrated in the nervous tissue itself, perhaps also on the surface of the convolutions.

The whole head appears swollen. The colour of the skin of the face is quite peculiar: it has lost its freshness, it has become serous. There is a heaviness in the eyelids; the eye is dead, void of expression. The globe of the eye projects behind the lids; the lids are slightly swollen; the eyebrows are moist; the head is bent upon the chest; the patient's attitude is heavy; he answers only yes, or no; his urine passes involuntarily; the tension of general paralysis is wanting. You observe no hesitation in his speech, nothing in his ideas that reveals ambitious exaggerations or conceptions.

Consider all these symptoms in the aggregate, and you will arrive at a collective phenomenon. This phenomenon is a state of stupefaction, of moral numbness. Thus modern observers are inclined to admit, as a constant thing in stupidity, a serous collection, even œdema of the brain.

Serous collections always announce themselves by some false appearance of a comatose state. This occasionally calls to mind serous apoplexy. The serous apoplectiform condition is frequently met with in general paralysis. It is announced by transitory paralysis of an eyelid, an arm, a leg, remarkable for disappearing in a few days.

In a hydrocephalic patient, properly so called, there are indications always sure by which the presence of a serous collection may be known: vomiting, dulness, mark the progress of the evil; the dilatation of the pupils, strabismus, paralysis of the eyelids, piercing cries, extreme slowness of the pulse, confirm it. But in the hydrocephalus of the insane, all often becomes doubt and uncertainty. In many patients we meet after death, serous collections, even considerable, that we were far from suspecting.

### Sixteenth Lecture.

*Cerebral softening. A patient affected with general paralysis.*—This patient is about thirty years old; he has been here a few months. You recognise his disease at a glance; that silly look, that uneasy bearing, cannot deceive you. Make him talk, and you will observe the hesitation of his speech; make him move, and you will see the uncertainty of his movements. Nothing so strange as his discourse: he talks of his strength, of the number of languages he knows—Russian, Danish, Spanish,—of his beautiful children, his young wife, the sums he has won. It is among patients of this category that you must seek for crebral softening. It is not found in all cases of general paralysis, but it is found exclusively in this affection.

By what sign can we recognise this organic lesion? The difficulty is great. If I consult my own observations, I discover in general paralysis and other paralyses, that which reveals to me that the cerebral substance is undergoing decomposition. This is a permanent, ascending, progressive paralysis. It is not the apoplectiform paralysis, but something resembling it. Ideas of grandeur, of exaggeration, the puerile aspect, which remind us of drunkenness, are not signs that indicate exclusively softening. They belong rather to an irritation of the grey substance, a work of decomposition that is preparing. The most characteristic marks of this state are clearly defined paralysis.

*Cadaveric phenomena.*—It is almost always the cortical substance that we find softened in the insane: this may be either the deep or the superficial layers. Sometimes there is softening of the white substance; but this alteration rarely affects the white substance exclusively; sometimes the white and grey substances are softened together. The parts most frequently affected are, in my opinion, the parietal regions, next the frontal. Sometimes the softening invades the upper median border of the hemispheres. It is rare to find it affecting the inner median surface of the hemispheres. Sometimes we see in the insane softening of the optic thalami, of the corpora striata, of the cerebellum.

In the dead body we recognise cerebral softening by—1, the abnormal aspect of the altered part; 2, the want of consistency of the cerebral substance; 3, changes in the intimate structure revealed by the microscope.

1. The grey substance acquires an ashy hue, greenish, sometimes violet, or yellowish, livid, rosaceous, or brownish; or it may be of a striking white.

2. The substance gives way under the slightest touch; it turns to pap, a semi-fluid element, easily taken up by the edge of the scalpel. The softening usually occupies a large extent. It is rarely an isolated condition; it is associated with serous collections, vascular engorgements, adhesions, thickening of the arachnoïd.

3. MM. Vogel and Gluge, and Pool of Amsterdam, have communicated interesting observations on the microscopic appearances. There have been found: capillary engorgement; extravasations of blood; inflammatory fibrinous products; nucleated cells; fatty globules; cumuli of red substance. These researches were made on subjects not insane. My investigations have been made on the sane and insane. I have compared the conditions observed in the two classes. My results differ from those of the microscopists I have named. I have not found traces of an inflammatory state: no fibrinous coagula, no inflammatory corpuscles, no islets of red matter.

The grey substance of a maniac seen under 400 degrees shows opaque nucleoli, tolerably regular in form, but irregularly distributed, soluble in ether, showing their fatty nature. I have found the same in bodies of persons not insane. They must not therefore be considered as a morbid result. The rest of the field seems formed of a cellular, granular web. It is in this web that the morbid histological phenomena take place.

If the cerebral substance is simply congested. you will perceive an infinity of cells, offering the appearance of a piece of Florence marble.

If the congestion has passed to the state of softening, you will have the same elements, but modified. In this case, the whole field presents a surface composed of these cellules. They are very irregular in their disposition, which may depend upon the traction the cerebral substance has undergone in charging the field. These cellules are polygonal, and have a visible nucleus. Each has usually but one; many are empty; and it is easy to detect here and there free nuclei. The cellules appear heaped together. At different points are remarked fatty cellules, recognised by their greater size and transparency. In a few points we discover blood-globules, but larger than usual, distended.

With great care, at a lower power, I have sometimes met with capillaries; they were gorged with deformed blood-globules. These capillaries were found at the surface of the cortical substance; deeper towards the white substance I could not distinguish them.

Such are the lesions which may be proved experimentally; but there is in this disease a complete series of phenomena, of which we cannot form an idea without the aid of the imagination and of reasoning. It is sometimes permitted to us to extend our judgment beyond the limits imposed by our senses; there are demonstrations, interpretative facts, which result from the collective examination of many facts, which, taken singly, are sometimes without value.

*Intimate phenomena.*—We may figure to ourselves the succession of the phenomena which characterise the formation of cerebral softening in the insane thus:—First, an excitation of the passions, of the ideas; a stimulation caused by the abuse of alcohol, or otherwise. A constant call of the circulating fluids into the capillaries. Distension of the capillaries. Engorgements. Stagnation of the fluids in these vessels. A serous transudation into the organic areolæ. An accumulation of serous fluids into the tissue of the pia mater. A penetration of these fluids into the grey substance of the brain, effected through the channels which give passage to the capillaries connecting the pia mater with the cortical substance. Then, the deformation of the primitive cellules. Considerable distension of these cells. Displacement of their nucleoli.

Clearly the nucleoïd cells found in softening are not new formations; they are the cells of the fundamental tissue of the grey substance. But they may exist ten times bigger than in the normal state. The reason is, that in softening, a serous fluid, escaped from the vessels, has penetrated them, and caused the distension. It is a true imbibition. In my opinion there is in the softening of the insane a maceration of the cerebral substance, a distension and a rupture of the primitive cells.

Let us dwell a moment upon what I have called the channels which transmit the capillaries running from the pia mater into the grey substance. These channels have attracted no attention; they are only discovered by a lens; they are, on a small scale, to the capillaries of the cortical substance what the canals of the liver of the capsule of Glisson are to the vessels of the vena porta, the arteries and biliary ducts. Myriads of capillaries, visible to the naked eye, in cases of stasis or inflammation, quit every point of the pia mater and dip into the cortical substance. It is by these vessels, which have not anastomosed, that the pia mater is made adherent to the grey substance of the convolutions. In cases of congestion these vessels acquire such a volume as to be distinguished by the naked eye.

Thus we may easily understand that in cases of serous collection between this covering and the convolutions, a road may be opened into the intimate tissue of the cortical substance, alongside the vessels. This infiltration produces maceration of the central substance.

A result little known, yet of great importance, is the extreme aptitude of the cerebral substance to be easily penetrated by foreign liquids. It may be compared in this respect to a sponge. Fred. and Herm. Nasse have shown that softened brains are much less easily penetrated by water than healthy brains.

When I say that general paralysis may exist without appreciable softening, I do not wish to utter an absolute dogma. The organic detritus may no doubt exist when our means of investigation cannot discover it.

Another remark: In every softening there is not paralysis.

I am anxious to say that the entire pathological state of this alteration is not summed up in congestion or in serous exaltation. What proves this is, that hyperæmia, sometimes considerable in mania and melancholy, rarely leads to softening. Cerebral softening is not a normal termination of the congestion in the insane. It is the same with stupidity which offers some analogy with the symptoms of softening, and which, viewed as an anatomical lesion, presents another serous infiltration. And yet in stupidity the cerebral tissue rarely passes into softening.

There is therefore something at the bottom of general paralysis, of the chief tactile alteration to which it is united, an obscured point, a boundary hitherto impassable.

Must we then admit different kinds of softening? I hesitate not to answer in the affirmative.

There is an acute softening, a chronic softening. It is the latter which is found in the insane. I believe that there are anæmic softenings.

Let us add that the causes which debilitate the organism are commonly hurtful to patients affected with this disease, and that the analeptic régime tends to prolong their days.

*Opacity of the arachnoïd thickening.*—In many cases the arachnoïd is much altered. Part injections of this membrane are not frequent unless the patient was much exalted in the domain of ideas, or of a very sanguine temperament. The most frequent appearance is a greyish white thickening. It may also present milky spots, and striæ. In some rare cases we find vitriform masses between the membranes. These alterations are principally found on the hemispherical surfaces, on the cranial layer, and not on that which covers the falx cerebri. They are rarely seen at the base of the organ; sometimes they are confined to one hemisphere; most frequently they extend to both. This condition especially belongs to chronic cases. It is really an isolated alteration. When isolated arachnoïdeal thickening is present, it is symptoms of compression that are observed There is however absence of paralysis of the limbs unless the thickening be considerable or accompanied by sanguineous effusions. If false membranes have formed between the meninges, they most commonly determine convulsions, alternating with a soporous state and transitory paralysis.

I recognise four morbid conditions, proceeding from the same source, which, in a diagnostic point of view, demand a long practical experience. These are, injection of the meninges, serous collections, chronic thickening of the membranes, cerebral softening. There exists between all these an affinity of origin, and a similitude of form. They all lead to obliteration of the intellectual acts. But an attentive observer may distinguish the individual character of each. Thus a disorder simulating a marked degree of drunkenness, corresponds more particularly to a fluxion of the meninges, especially of the pia mater, and of that of the cortical substance.

The presence of serosity causes different shades of dulness, stupor, inertia, coma.

Thickening, the retractation of the arachnoïd compressing the brain, causes to a certain degree a diminution of intellectual energy, but leaves considerable freedom of motion.

Softening affects more directly motility, and paralysis more directly the motor influx, especially of speech.

I cannot say often enough how important it is to acquire two general notions. That which teaches us to know a brain disordered only in its functions, not in its structure; that which enables us to recognise a brain diseased in its anatomical elements.

Clearness, neatness of expression, absence of disorder in the connexion of ideas, demonstrate that there exists no anatomical lesion: this is only known by observation, failure of the phrenic acts. To this, we must add the elements of appreciation. It is known that alterations of tissue are rare in melancholy, ecstasy, delirium, folly.

It is in two forms of alienation that doubt always arises: dementia, mania.

Conviction springs up when we see signs of compression of cerebral destruction. This certainly is wanting so long as the signs which belong to paralysis are absent; *i. e.*, paralysis in the formation of words, of the intelligence, of memory, of movements, of prehension, of locomotion. What adds to the clearness of the diagnosis is the reunion of paralysis, of convulsions, of ebrious ideas.

*Meningo-cerebral adhesions.*—I have no settled opinions concerning adhesions between the arachnoid and the dura mater. It would be a grave error to conclude that they are the result of inflammation.

*Cerebral induration.*—It is considered that induration is met with in 25 out of 100 insane. It is most frequently found in chronic mania, in dementia, and in maniacal epileptics; also in general paralysis with softening. I think I have observed that this alteration is most commonly seen at the base of the brain and in the external walls of the lateral ventricles. More than once I have found the pons Varolii so hard as to be nearly crepitant under the knife. Induration of the olivary bodies is not at all rare. It chiefly affects the grey matter; but may affect the white also.

The intimate nature of the pathological alteration is difficult to determine. My microscopical investigations have taught me nothing precise.

Are there any symptoms which permit us to recognise induration in the living subject? Hitherto they have not been pointed out.

*Cerebral hypertrophy and atrophy.*—I have often observed hypertrophy, especially in maniacs. In these cases the convolutions are so compressed against the skull, that they are sometimes only traced by lines. This state is culiar to congestional mania.

Atrophy may be general or partial. Partial—it is often confined to a series of convolutions. General—the brain has diminished in volume, and is found separated from the inner table of the cranium, as Gall was the first to observe. It has been supposed that atrophy was most common in the frontal region, and I have several times verified this observation. Parchappe says it is met with in 16 out of 100 cases. He calls it cerebral marasmus.

This condition belongs especially to chronic dementia. I think also it belongs to melancholy.

*Vices of conformation of the skull and brain.*—These are chiefly seen among idiots.

*Of the anatomical alterations of the abdominal and thoracic viscera.*

M. Parchappe, the man who has best investigated this subject, calculates that out of 1000 insane patients, 423 present after death lesions of the cerebro-spinal system; 262 lesions of the digestive canal; 140 in the respiratory system.

A. *Affections of the alimentary canal.*—I have found thickening of the walls of the stomach. Scirrhous induration of the pylorus, inflammation, ulceration, softening. But in most instances these have appeared to me independent of the mental malady. I have observed in suicide inflammation of the intestinal mucous membrane, once in a case where there was no cerebral disease. Some physicians have attributed great importance to the pathological state of the intestines in melancholy.

2. The ideas of Esquirol as to the displacement of the colon have been confirmed; in the insane we do indeed sometimes find this intestine lodged in the true pelvis.

3. Inflammations of the peritoneum, the omentum adhering to the mesentery, and this latter to the abdominal wall, &c.

4. In suicide, considerable abdominal lesions are observed.

B. *Affections of the liver and spleen.*—It is not rare to find red spots upon the liver. It is found crepitant, often gorged with blood, and exhibiting traces of inflammation. Alterations in the liver are frequent in drunkards. But I have examined the bodies of persons dead from delirium tremens without finding any appreciable lesion of the liver.

I remember a case of joyous mania which presented to me an enormous distension of the spleen which contained a very black blood. I asked myself if this case did not support the opinion of some of the ancients who placed gaiety in the spleen and anger in the liver.

In the melancholic, the suspension of respiration—*i. e.*, its performance at long intervals and imperfectly, explains in a great measure the frequent presence of engorgements of the system of the vena portæ and especially of the liver and spleen. Very often the mesenteric veins are found loaded with black blood.

In dementia we sometimes find enormous distensions of the urinary bladder.

I have recognised disease of the ovaries, after a violent delirium accompanied by hysterical symptoms. The menstrual suppression, so frequent in insane women, should point to the conjecture that the ovaries are frequently affected.

C. *Affections of the lungs.*—In estimating the pathological condition of the lungs, we must bear in mind the influence of the variations of temperature to which the insane have been exposed, their cries and vociferations, disease of the pneumogastric nerve, insufficient food, spermatic evacuations, the use of cold douches, a strumous constitution.

Pulmonary tuberculosis is frequent amongst the insane. It has appeared to me to have a direct relation to mental alienation. Sometimes this is allied with meningeal or cerebral tuberculosis. A tuberculous condition of the substance of the brain has been denied. But this is a serious error. I have observed it frequently; and I may call in the testimony of my colleague, Professor Mareska.

I have observed *gangrene* of the lungs, and this has been exclusively in cases where the patients refused to eat. This has been subsequently confirmed. In fasting madmen also gangrene of the intestinal mucous membrane has been found. There is evidently a disordered hæmatosis in these patients.

The symptoms of pulmonary gangrene are announced in too clear a manner to allow a practised eye to be deceived. There is no disturbance in the mechanical phenomena of respiration. It is in the blood that a profound alteration exists. The general colour of the skin indicates this; it becomes yellow, brownish, the colour of beer. The conjunctiva puts on a bluish tint. A remarkable decomposition is observed in the features. Red spots and swellings appear in different parts of the body. The breath exhales a horrible fœtor. Sometimes a slight cough appears: the patient at first expectorates frothy mucus, next the mucus is streaked with blood; then this is replaced by a brownish sanies of extreme fœtidity.

We must not, however, conclude that gangrene of the lungs takes place in every case of refusal to eat.

*Affections of the heart.*—These are not infrequent among the insane. You will not lose sight of the fact that the heart plays a great part in the moral acts. The cries and continual groans of the patients disturb the action of the heart, and drive the blood back upon the right cavities; sorrow, muscular prostration, render the dilatation of the chest imperfect, and oppose an obstacle to the circulation of the blood.

*Conclusion. General inductions.*—The anatomical diagnosis presents no inconsiderable difficulties in its application to mental diseases. But I will endeavour to sum up what science permits as to formulize in this respect. Every kind of phrenopathy may present cadaveric lesions; but these may also be found in other diseases, in which their signification is altogether different.

*Melancholy.*—If melancholics die accidentally during the phrenalgic state, they present for the most part no trace of organic alteration either of the brain or meninges. The solidity of the cerebral substance, a venous turgescence, a slight sinking in, a slight serous collection, are the only phenomena observed inside the cranium.

If the disease is protracted beyond the ordinary term of cure, if it become insensibly associated with debility of the functions of the understanding, we may admit that a change has taken place in the organic change of the encephalon, an opacity of the arachnoïd, a hyperæmia of the pia mater, and most frequently an inter-membranous serous effusion.

In melancholy, more than in any other kind of mental disease, autopsy leads to the discovery of visceral lesions, of engorgements of the vena portæ, inflammations of the peritoneum, affections of the chest; but, in the majority of cases, these are the result of the disease or of fortuitous circumstances.

*Ecstasy.*—It is rare to see patients affected with ecstasy succumb to the disease: we must conclude that it is exempt from any disorganizing condition; and that in this affection as in melancholy, as in the generality of manias, the cerebral disorder is simply functional.

*Mania.*—When mania is accompanied by injection of the conjunctiva, great heat in the scalp, we must conclude that there is cerebro-meningeal hyperæmia, but not inflammatory or disorganizing. It is the expression of a functional exaltation, of an orgasm communicated to the vascular system.

When mania is characterized by a great influx of will, by cries, tumult, agitation, the encephalon is gorged with blood. We often find in patients who have vociferated much, congestions of the pia mater and sub-arachnoïd ecchymosis: they usually exist in the parietal and temporal regions.

If the patient die accidentally in the course of a tranquil mania, if he have preserved intact conception, memory, the affective sentiments, autopsy hardly ever reveals the slightest organic alteration. This is also true of manias which break out periodically; in the intervals, the brain presents nothing abnormal.

When mania is complicated with epilepsy, the head is congested at every fit; often there are found sub-arachnoïdeal ecchymoses of the brain itself, of the cortical and medullary substance, indurations of the pons, of the medulla oblongata.

When mania, after having lasted several months, passes insensibly into a state of intellectual prostration, we can no longer say with certainty if there exist in the patient one or other of the anatomical alterations I have pointed out. If the symptoms of mania go on diminishing, and those of dementia increasing, we may be almost sure that a morbid organic change has been wrought. Most frequently we then meet with congestion of the cortical substance, of the pia mater, thickening of the arachnoïd, rarely with softening.

Sometimes in maniacs we find hyperæmia, opacity of the arachnoïd; we must conclude that these lesions mark a serosity unusual in this disease.

I do not hesitate to lay it down as a principle, that in the majority of cases mania excludes appreciable organic lesions.

*Folly.*—I cannot say what is the condition of the brain in incendiary, homicidal, fasting suicidal madmen. If the disease has been of long duration, a morbid condition of the thoracic and abdominal viscera is frequently recognised.

*Delirium.*—The same uncertainty pervades the entire series of phrenopathies we have comprised under the name of delirium.

*Dementia.*—It is especially in dementia that we must expect to find anatomical lesions of the brain. Amongst all the phenomena which announce these lesions, the principal are, subtraction, nullity, volition of the cerebral and muscular acts. These are occasioned by compression, destruction, or even by irritation of the cerebral pulp.

In dementia, more than in any other phrenopathy, we may expect thickening, shrinking of the arachnoïd, infiltration, vascular engorgement of the pia mater, a modification in the vascular state and texture of the neighbouring convolutions.

But we cannot always say that there is, or that there is not softening.

We may often affirm the existence of a serous collection.

As to cerebral induration, the little certainty as to its symptoms does not allow us to conjecture its presence.

It is essential to remember that dementia is not invariably linked with an organic state of the brain; this disease is often completely independent of such a state.

Such is the character of the greater number of pure primitive dementias. In senile dementia, in that form which follows immediately upon a strong moral commotion, in dementia the result of great misery, in that which is connected with spermatorrhœa, the cadaveric inspection usually reveals no morbid state whatever. I except serous accumulations, a state of discoloration of the grey substance, falling in of the brain. But I repeat it; it is not always permitted to us to say, I shall find in such a patient a discoloration, a shrinking, an inter-membranous hydrocephalus.

---

Here we terminate the phenomenological part of mental diseases. We shall next discuss the etiology of these affections.

---

